# Dynamics of Antibody Responses after Asymptomatic and Mild to Moderate SARS-CoV-2 Infections: Real-World Data in a Resource-Limited Country

**DOI:** 10.3390/tropicalmed8040185

**Published:** 2023-03-23

**Authors:** Naruemit Sayabovorn, Pochamana Phisalprapa, Weerachai Srivanichakorn, Thanet Chaisathaphol, Chaiwat Washirasaksiri, Tullaya Sitasuwan, Rungsima Tinmanee, Chayanis Kositamongkol, Pongpol Nimitpunya, Euarat Mepramoon, Pinyapat Ariyakunaphan, Diana Woradetsittichai, Methee Chayakulkeeree, Pakpoom Phoompoung, Korapat Mayurasakorn, Nitat Sookrung, Anchalee Tungtrongchitr, Rungsima Wanitphakdeedecha, Saipin Muangman, Sansnee Senawong, Watip Tangjittipokin, Gornmigar Sanpawitayakul, Cherdchai Nopmaneejumruslers, Visit Vamvanij, Chonticha Auesomwang

**Affiliations:** 1 Division of Ambulatory Medicine, Department of Medicine, Faculty of Medicine Siriraj Hospital, Mahidol University, Bangkok 10700, Thailand; 2 Department of Nursing, Faculty of Medicine Siriraj Hospital, Mahidol University, Bangkok 10700, Thailand; 3 Division of Infectious Diseases and Tropical Medicine, Department of Medicine, Faculty of Medicine Siriraj Hospital, Mahidol University, Bangkok 10700, Thailand; 4 Siriraj Population Health and Nutrition Research Group, Department of Research Group and Research Network, Faculty of Medicine Siriraj Hospital, Mahidol University, Bangkok 10700, Thailand; 5 Center of Research Excellence on Therapeutic Proteins and Antibody Engineering, Department of Parasitology, Faculty of Medicine Siriraj Hospital, Mahidol University, Bangkok 10700, Thailand; 6 Department of Parasitology, Faculty of Medicine Siriraj Hospital, Mahidol University, Bangkok 10700, Thailand; 7 Department of Dermatology, Faculty of Medicine Siriraj Hospital, Mahidol University, Bangkok 10700, Thailand; 8 Department of Anesthesiology, Faculty of Medicine Siriraj Hospital, Mahidol University, Bangkok 10700, Thailand; 9 Department of Immunology, Faculty of Medicine Siriraj Hospital, Mahidol University, Bangkok 10700, Thailand; 10 Division of Ambulatory Paediatrics, Department of Paediatrics, Faculty of Medicine Siriraj Hospital, Mahidol University, Bangkok 10700, Thailand; 11 Department of Orthopaedic Surgery, Faculty of Medicine Siriraj Hospital, Mahidol University, Bangkok 10700, Thailand

**Keywords:** antibody response, booster, COVID-19 vaccine, neutralizing antibody, waning of immunity

## Abstract

The dynamics of humoral immune responses of patients after SARS-CoV-2 infection is unclear. This study prospectively observed changes in anti-receptor binding domain immunoglobulin G (anti-RBD IgG) and neutralizing antibodies against the Wuhan and Delta strains at 1, 3, and 6 months postinfection between October 2021 and May 2022. Demographic data, clinical characteristics, baseline parameters, and blood samples of participants were collected. Of 5059 SARS-CoV-2 infected adult patients, only 600 underwent assessment at least once between 3 and 6 months after symptom onset. Patients were categorized as immunocompetent (*n* = 566), immunocompromised (*n* = 14), or reinfected (*n* = 20). A booster dose of a COVID-19 vaccine was strongly associated with maintained or increased COVID-19 antibody levels. The booster dose was also more strongly associated with antibody responses than the primary vaccination series. Among patients receiving a booster dose of a mRNA vaccine or a heterologous regimen, antibody levels remained steady or even increased for 3 to 6 months after symptom onset compared with inactivated or viral vector vaccines. There was a strong correlation between anti-RBD IgG and neutralizing antibodies against the Delta variant. This study is relevant to resource-limited countries for administering COVID-19 vaccines 3 to 6 months after infection.

## 1. Introduction

Between the emergence of coronavirus disease 2019 (COVID-19) in late 2019 and the beginning of August 2022, over 582 million cases of infected patients and approximately 6.4 million deaths have been reported [1]. The primary cause was severe acute respiratory syndrome coronavirus 2 (SARS-CoV-2). The precise and rapid diagnosis of this viral infection has been globally accepted as the key to successful treatment outcomes. Comprehensive vaccine administration is one of effective methods to prevent the progression of COVID-19, lower the mortality, and control disease transmission [2,3,4]. In many countries, a mRNA vaccine (Pfizer–BioNTech BNT162b2) was the first COVID-19 vaccine. The vaccine was given emergency use authorization by the United States Food and Drug Administration on 11 December 2020. The organization officially approved the vaccine on 23 August 2021 [5].

In Thailand, COVID-19 vaccines first became available in February 2021. The main types were inactivated vaccines (CoronaVac and Sinopharm) and an adenovirus vector vaccine (ChAdOx1 nCoV-19, AstraZeneca). A small batch of the BNT162b2 (Pfizer–BioNTech) vaccine was initially delivered in July 2021, some of which was retained for research. The mRNA-1273 (Moderna) vaccine started being delivered in November 2021. This meant that Thais could increasingly access mRNA COVID-19 vaccines. However, by late 2021, many Thais had already received a primary series based on the inactivated and adenovirus vector vaccines available from February 2021. Further vaccination with the mRNA vaccines was encouraged even if individuals had already been infected because the inactivated and adenovirus vector vaccines did not provide robust immunity [6,7]. People that are infected with SARS-CoV-2 develop natural immunity which consists of humoral and cellular immunity. The antibody responses can be detected within the first week after the onset and last only a few weeks to several months before declining [8,9,10,11,12]. Neither the past infection nor vaccination provided lifelong protection against reinfection incidence. Booster vaccination doses were, therefore, considered a critical strategy to maintaining high levels of immune response and preventing reinfection at that time (around 2020–2021) [11,13].

Although the advantages of COVID-19 vaccination seem convincing, some patients hesitate or refuse to receive vaccines, either as primary series or booster doses. Their reluctance stems from the current lack of scientific data on the long-term effects of the COVID-19 vaccines [14]. The levels of antibodies (anti-spike immunoglobulin G [IgG], anti-receptor binding domain [RBD] IgG, anti-nucleocapsid IgG, and neutralizing antibodies) have become measurable indicators and have been widely reported [15,16]. Nevertheless, there was still limited evidence that patients receiving at least one booster dose after SARS-CoV-2 infection have stronger immunity than those without a booster dose around mid to late 2021.

Siriraj Hospital, Thailand, implemented home isolation and hospital-affiliated hotel isolation (“hospitel”) management strategies for patients with asymptomatic and mild to moderate COVID-19 in the second trimester of 2021. Longitudinal data on the humoral immune responses of patients with SARS-CoV-2 infections can be helpful and may be associated with some clinical data. The results may materially affect vaccination recommendations and patients’ judgments on whether to receive a booster dose, especially in a resource-limited country. This study aimed to evaluate the dynamics of antibody responses for various vaccination statuses during the 6 months following a COVID-19 illness. Moreover, we examined the correlation between the responses of anti-RBD IgG and neutralizing antibodies against the Wuhan and Delta (B.1.617.2) variants of SARS-CoV-2. This arises from the concern over whether neutralizing antibody against the Delta strain may be predicted by the levels of anti-RBD IgG and neutralizing antibody against the Wuhan strain.

## 2. Materials and Methods

### 2.1. Study Design and Participants

This prospective cohort study enrolled patients who had recovered from COVID-19 infection and had been registered for care under Siriraj Hospitals’ home isolation and hospitel systems. Eligible patients had to be 18 years old or older with asymptomatic infection or mild to moderate illness of COVID-19, per the National Institutes of Health guidelines [17]. The diagnosis of SARS-CoV-2 infection was confirmed with real-time reverse transcription polymerase chain reaction. All participants were infected between August and November 2021 (during the Delta wave of COVID-19). They were enrolled at 1 month or 3 months after symptom onset and were followed up until 6 months after the onset. The exclusion criterion was patients who visited only once during the 1-month postinfection period. Before the research began, the Ethics Committee of the Siriraj Institutional Review Board approved its protocol (Si 732/2021; 20 September 2021).

**Figure 1:**
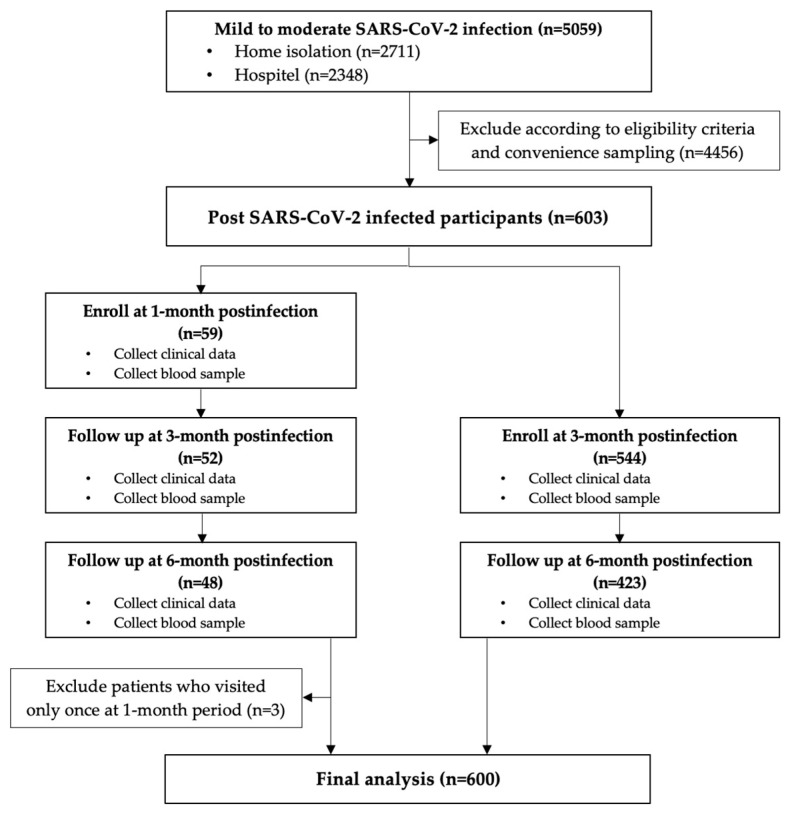
Flowchart of patient enrollment.

**Figure 2:**
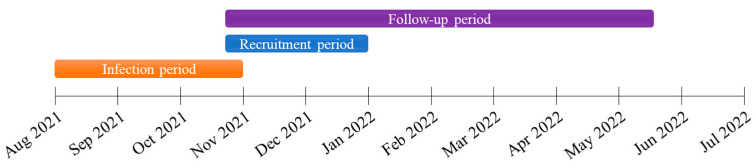
Timeline of the study.

Flowcharts of the patient enrollment and follow-up processes are presented in Figure 1 and Figure 2. Eligible subjects were recruited at Siriraj Hospital between 26 October and 27 December 2021. Before entering the study, participants signed an informed consent form and were advised that they could withdra at any time. The project site initially recorded demographic data, clinical characteristics, and baseline parameters related to immunity. Blood samples and clinical data were collected at the 1-, 3-, and 6-month visits to evaluate participants’ antibody responses. At each time point, all blood chemistry data and three antibody responses including anti-RBD IgG, neutralizing antibodies of SARS-CoV-2 against Wuhan and Delta strains, were investigated using the same blood sample. Visits were scheduled from symptom onset or (for patients without symptoms) the positive diagnosis. The last follow-up visit was on 17 May 2022. Per the public health policy, subjects could choose any COVID-19 vaccine type after infection. Some participants had SARS-CoV-2 reinfections within 6 months after their first episode. For analysis purposes, these participants were categorized into a reinfection group.

According to the Centers for Disease Control and Prevention, immunocompromised patients in our study were those who had a decreased immune system as a result of a medical illness or a therapy for a condition. This includes people with cancer who are receiving chemotherapy or those who have received a solid organ transplant, such as a kidney or heart, and are taking medication to maintain their transplant. Others include those who were infected with the human immunodeficiency virus (HIV). Patients who did not meet the conditions listed above were classified as the immunocompetent group [18].

### 2.2. Antibody Responses and Clinical Data

IgG antibodies against the RBD of the S1 subunit of SARS-CoV-2 spike proteins (SARS-CoV-2 IgG II Quant for use with ARCHITECT I system; Abbott Laboratories, Abbott Park, IL, USA) were detected using chemiluminescent microparticle immunoassay (CMIA). The results are presented in arbitrary units (AU/mL). Levels ≥50 AU/mL were considered positive. The analytical measurement range was 21.0–40,000.0 AU/mL, and, with dilution, it was possible to increase it to 80,000.0 AU/mL. According to the WHO international Standard concentration, the result must be converted to the binding antibody unit per mL (BAU/mL) by multiplying with 0.142, which was provided by the manufacturer [19].

A Surrogate Virus Neutralization Test (sVNT) was used for determining the level of the neutralizing antibody against SARS-CoV-2 virus including Wuhan and Delta (B.1.617.2) strains. This assay was developed by BioDesign Innovation Center, Department of Parasitology, Faculty of Medicine Siriraj Hospital, Mahidol University, Nakhon Pathom, Thailand. sVNT is based on the interfering of neutralizing antibody to binding of horseradish-peroxidase-conjugated (HRP-conjugated) RBD protein and angiotensin-converting enzyme 2 (ACE2). The assay was validated with 100 SARS-CoV-2 infected patients and 100 healthy persons using the Plaque Reduction Neutralization Test (PRNT) utilizing the SARS-CoV-2 virus, Wuhan and Delta variants as the comparator. It was found that established sVNT showed the correlation to PRNT with nearly 90% agreement. The ROC curve showed 30% as an optimum cut-off for sVNT. (Data are not shown.) During the time of the study, this testing of two strains (SARS-CoV-2 virus, Wuhan and Delta variants) was accessible. The individual HRP-conjugated RBD protein of Wuhan and Delta variants (Fapon Biotech, Dongguan, China) were used as the probe in individual reaction. The sample and control tests were pre-incubated with HRP-conjugated RBD protein which renders the binding of neutralizing antibody to the target. Then, the mixture was applied to the well containing Streptavidin bound with Biotin-conjugated ACE2. Free HRP-conjugated RBD protein was bound to ACE2. While HRP-conjugated RBD protein binding the neutralizing antibody in the supernatant was removed with a washing step. Tetramethylbenzidine (TMB) substrate and stop solution were added. Finally, the optical density absorbance was measured using a spectrophotometer at 450 nm. The inhibition rate was calculated through this formula:

$$\mathrm{Inhibition}=\left(1-\frac{{\mathrm{OD}}_{450}\;\mathrm{sample}}{\begin{array}{c} {\mathrm{OD}}_{450}\;\mathrm{negative}\;\mathrm{control} \end{array}}\right)\;\times \;100\%$$

Patient demographic data, anthropometric data, comorbidities, medication use, and vaccination histories were obtained from interviews and medical records.

### 2.3. Statistical Analysis

Categorical variables are presented as frequencies and percentages. Continuous variables with a normal distribution are reported as the means ± standard deviations, and those with a non-normal distribution are reported as the medians with interquartile ranges. The independent sample *t*-test or the Mann–Whitney U test or analysis of variance (ANOVA) was used to evaluate the differences in the continuous variables of patient groups. The chi-square or Fisher’s exact test was used for categorical variables. Univariable and multivariable logistic regression models were employed to explore the associations between clinical parameters and antibody responses. The regression results are presented as crude odds ratios (cORs) and adjusted odds ratios (aORs). Statistical significance was set at *p* < 0.05. The correlation of anti-RBD IgG and neutralizing antibodies was determined using Spearman’s correlation analysis, with a strong correlation defined as r > 0.7. All statistical analyses were performed using SPSS software version 28.0 (IBM Corp, Armonk, NY, USA) and GraphPad Prism, version 9 (GraphPad Software Inc., San Diego, CA, USA).

## 3. Results

### 3.1. Baseline Characteristics

Although there were 5059 patients with SARS-CoV-2 infection, 4456 patients were ineligible for enrollment because of age or their inability or unwillingness to participate in the study. The remaining 603 adults volunteered as research subjects. However, three patients were subsequently excluded from our analyses because they visited the study site only once (at the 1-month follow-up). The other 600 participants underwent clinical, metabolic, and immune assessments at least once at 3- and 6-month visits. There were 56, 596, and 471 participants at 1-, 3-, and 6-month visits, respectively, as shown in Figure 1. A total of 1126 blood samples were collected for the analysis.

The participants’ baseline demographic and clinical characteristics are summarized in Table 1. The data are categorized into three groups including immunocompetent (n = 566), immunocompromised (*n* = 14), and reinfection (*n* = 20). Immunocompromised patients were those with human immunodeficiency virus infection (*n* = 12) and post kidney transplantation (*n* = 2). Participants in the reinfection group had a normal immune status. Mean ages were 45.8 ± 14.6 years (immunocompetent group), 44.3 ± 10.3 years (immunocompromised group), and 37.1 ± 10.7 years (reinfection group), *p* value = 0.029. The majority of subjects in each group were women (61.5%–65.0%, *p* value = 0.961). Body weights at the symptom onset were comparable: 66.7 ± 15.5 kg (immunocompetent group), 60.5 ± 13.4 kg (immunocompromised group), and 74.4 ± 17.7 kg (reinfection group), *p* value = 0.029. Body mass indexes (BMI) at the symptom onset were 25.4 ± 5.0, 22.3 ± 4.8, and 27.1 ± 5.1 kg/m^2^, respectively, *p* value = 0.022. Body weights and body mass indexes at the 3-month postinfection visit were slightly higher than those at symptom onset. Regarding risk factors for progressive diseases in the immunocompetent group, 103 patients (18.2%) were ≥60 years of age, 87 patients (15.4%) had a BMI ≥ 30 kg/m^2^, and 254 patients (44.9%) had at least one comorbidity. In the immunocompromised group, there was one patient (7.1%) with age ≥ 60 years and one patient (7.1%) with a BMI ≥ 30 kg/m^2^. Almost half of reinfected patients had a BMI ≥ 30 kg/m^2^ (8; 40.0%). Most participants in the whole cohort had symptoms related to COVID-19 at onset. Forty-eight (8.0%) patients with moderate COVID-19 had a history of additional dexamethasone to treat deteriorating COVID-19 symptoms.

Approximately one-third of the immunocompetent patients (206; 36.4%) and the reinfected patients (6; 30.0%) were unvaccinated before infection. More than half of immunocompromised group were unvaccinated (8; 57.1%). The cycle threshold (Ct) was not markedly different among the three groups. Most immunocompetent patients (543; 95.9%) and all patients (100.0%) in the other two groups were treated with favipiravir. At the 3-month postinfection follow-up, over 80% of the 600 patients were fully vaccinated, the remaining were partially or unvaccinated. During the 3-month postinfection period, 307 (56.7%) immunocompetent patients, 11 (78.6%) immunocompromised patients, and 6 (30.0%) patients who had reinfection received a booster dose of the COVID-19 vaccine. Over half of the 600 subjects (310; 51.7%) received a booster dose between 3 and 6 months after infection. At the 3-month follow-up, there were no significant differences in the demographic data and clinical parameters of immunocompetent participants with any vaccination status (Table 2).

Patients without a booster dose within 3 months postinfection had a higher chance of reinfection (5.6%; 14/251) than those receiving a booster dose of any type during that period (1.9%; 6/324; cOR, 0.319; 95% CI, 0.121–0.843; *p* = 0.021), as shown in Supplementary Materials Table S1.

### 3.2. Factors Associated with Antibody Responses

Table 3 details the relationships between the levels of anti-RBD IgG and neutralizing antibodies against the two viral strains at the 3-month follow-up. Table 3 also shows the types of COVID-19 vaccines used by participants who were fully vaccinated at the onset of infection (*n* = 158). There were six primary vaccination series: two doses of inactivated vaccine (CoronaVac or Sinopharm); two doses of adenovirus vector vaccine (AstraZeneca); one dose of inactivated vaccine and one dose of adenovirus vector vaccine (AstraZeneca); one dose of inactivated vaccine and one dose of mRNA vaccine (BNT162b2, Pfizer–BioNTech); one dose of adenovirus vector vaccine (AstraZeneca) and one dose of mRNA vaccine (BNT162b2, Pfizer–BioNTech); two doses of mRNA vaccine (BNT162b2, Pfizer–BioNTech).

For each series, the antibody levels of patients with and without a booster dose within 3 months postinfection were compared. Mostly, there were no significant differences between the two groups of patients in the levels of anti-RBD IgG and neutralizing antibodies for all primary series. The exception was the level of neutralizing antibody against the Wuhan strain from those who received a booster dose or did not, with regard to the primary series of two doses of inactivated vaccine (CoronaVac or Sinopharm).

The anti-RBD IgG levels of patients receiving a booster dose within 3 months postinfection were analyzed (*n* = 52). The effect of the mRNA booster following a primary series of two inactivated vaccines was significantly higher than the adenovirus vector booster, in terms of a higher level of 3-month anti-RBD IgG. The median anti-RBD IgG levels were 23,099 and 10,113 AU/mL, respectively (*p* = 0.002). This difference had not been demonstrated in the regimen boosted with mRNA following any primary series (median, 20,969 vs. 10 113 AU/mL; *p* = 0.125). The different levels of neutralizing antibodies at the 3-month follow-up were not statistically significant in each vaccine regimen. The details are shown in Supplementary Materials Table S2.

An analysis was made of the factors associated with antibody levels remaining steady or increasing between the 3- and 6-month follow-ups (*n* = 448). Age was substantially associated with reduced anti-RBD IgG levels from multivariable logistic regression analysis (aOR, 0.97; 95% CI, 0.95–0.99; *p* = 0.005) but not significantly associated with reduced anti-RBD IgG levels at 6 months (cOR, 0.99; 95% CI, 0.97–1.00; *p* = 0.062). However, age was not associated with neutralizing antibody levels. Another factor strongly associated with antibody levels being maintained or increased was the acquisition of a booster dose. The cOR of anti-RBD IgG of participants with no booster vaccine within 3 months but boosted between 3 and 6 months was 27.36 (95% CI, 12.07–62.02; *p* < 0.001), while the aOR was 29.89 (95% CI, 12.55–71.15; *p* < 0.001). The cOR of anti-RBD IgG of participants with two booster doses between 0 and 6 months was 25.30 (95% CI, 10.91–58.69; *p* < 0.001), while the aOR was 34.05 (95% CI, 13.88–83.55; *p* < 0.001). The aORs of the Wuhan- and Delta-strain neutralizing antibodies of those with no booster vaccine within 3 months but boosted between 3 and 6 months were 2.53 (95% CI, 1.37–4.68; *p* < 0.001) and 10.83 (95% CI, 5.56–21.09; *p* < 0.001), respectively. The aORs of the Wuhan- and Delta-strain neutralizing antibodies of those with two booster doses between 0 and 6 months were 2.26 (95% CI, 1.20–4.27; *p* < 0.001) and 11.55 (95% CI, 5.74–23.22; *p* < 0.001), respectively. The details are shown in Table 4.

### 3.3. Dynamics of Antibody Responses

Table 5 summarizes the median changes in antibody levels between 3 and 6 months postinfection of 18 patients with various COVID-19 vaccine regimens. Negative values in Table 5 signify decreasing anti-RBD IgG responses. Patients with two inactivated doses (primary series) and one AstraZeneca and one mRNA dose as boosters had significantly higher anti-RBD IgG levels than those with two inactivated doses (primary series) and two AstraZeneca booster doses (*p* = 0.020). However, there were no significant differences in the neutralizing antibody levels of each vaccine regimen against the Wuhan and Delta strains (*p* = 0.371 and 0.077, respectively).

**Figure 3:**
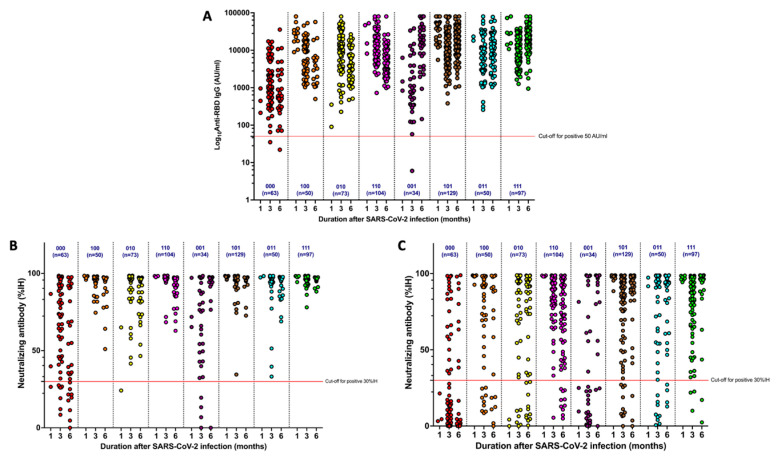
Dynamic changes of antibody responses for various regimens of COVID-19 vaccine (n, number of participants in each group). (**A**) Log_10_Anti-RBD IgG; (**B**) Neutralizing antibody against Wuhan strain; (**C**) Neutralizing antibody against Delta strain. 000, did not receive vaccine at any time; 100, received vaccine before the illness but did not receive after the illness; 010, received vaccine only within 3 months postinfection; 110, received vaccine before the illness and boost after the illness within 3 months; 001, received vaccine between 3–6 months postinfection only; 101, received vaccine before the illness and boost after the illness only at 3–6 months postinfection; 011, did not receive vaccine before the illness and received booster vaccine two doses at 0–3 months and 3–6 months postinfection; 111, received vaccine all the period (before the illness, 0–3 months postinfection, 3–6 postinfection).

Patient antibody responses were categorized into eight groups (Figure 3). The differences between the groups were vaccination status before SARS-CoV-2 infection, within 3 months postinfection, and from 3 to 6 months postinfection. In the scatter plots, “1” represents patients given at least one COVID-19 vaccine dose, while “0” refers to patients who did not receive any COVID-19 vaccine during the period. Anti-RBD IgG and neutralizing antibodies levels were lowest among patients who were not vaccinated before or after their COVID-19 illness. Receiving at least one booster dose during the postinfection period maintained anti-RBD IgG and neutralizing antibodies levels, especially between 3 and 6 months postinfection. In the case of patients who did not receive a booster dose between 3 and 6 months postinfection, anti-RBD IgG and neutralizing antibodies levels tended to decline. The details were demonstrated, separately, in three groups and all of participants as well as in eight groups of vaccination status, as shown in Supplementary Materials Table S3–S6.

**Figure 4:**
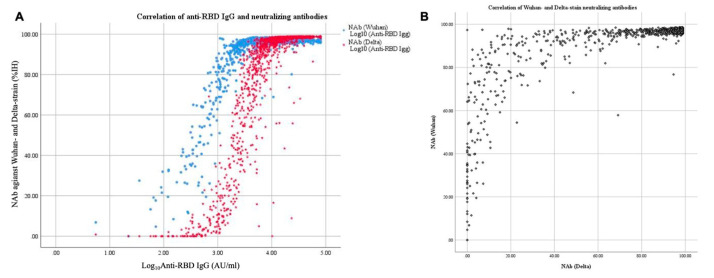
Correlations of antibody responses of post-SARS-CoV-2 infected participants (600 participants with 1126 blood samples). (**A**) Between anti-RBD IgG and neutralizing antibodies against Wuhan and Delta strains; (**B**) Between neutralizing antibodies against Wuhan and Delta strains.

Figure 4 shows the correlation of antibody responses from all 1126 blood samples. Log_10_Anti-RBD IgG was strongly correlated with neutralizing antibodies against the Delta strain (Spearman’s r, 0.91; *p* = 0.01). Neutralizing antibodies against the Wuhan strain were non-significantly correlated with both log_10_Anti-RBD IgG (Spearman’s r, 0.57; *p* = 0.01) and neutralizing antibodies against the Delta strain (Spearman’s r, 0.59; *p* = 0.01).

### 3.4. Reinfection

All of the reinfection occurred between January and April 2022 which was 3–6 months after the first infection onset. The Anti-RBD IgG and neutralizing antibodies level of patients suffering a second episode of SARS-CoV-2 infection within 6 months after their first infection (n = 16) are presented in Table 6. At 6 months postinfection, there was significant difference in the group of patients who had received a booster dose between their 3- and 6-month Anti-RBD IgG level (*p* = 0.021). Whether patients had received the booster dosage or not, neutralizing antibodies against the Wuhan and Delta strains were not statistically significant. However, this finding may result from an inadequate sample size. The regimens of vaccine in the reinfected patients were shown in Supplementary Materials Table S7.

## 4. Discussion

In this study, patients with a prior SARS-CoV-2 infection were recruited to evaluate the dynamics of their immune responses. Antibody responses usually occur within 1 to 3 weeks after infection and peak at weeks 4 and 5 [9,12,20,21]. The responses start to decline 2 to 3 months postinfection [21,22,23,24]. We examined the levels of anti-RBD IgG and neutralizing antibodies against two viral strains in adult patients with asymptomatic and mild to moderate SARS-CoV-2 infections during the 6-month postinfection period. Their correlations may provide useful direction for the practical application of the antibody test.

Following the start of the COVID-19 Delta wave in Thailand in mid-2021, the availability of the mRNA COVID-19 vaccine encouraged vaccine uptake due to the vaccine’s high efficacy. Nevertheless, some patients still insisted on being administered an adenovirus vector vaccine because it had fewer reported side effects than a mRNA vaccine. No immunocompromised patient from our study was classified as fully vaccinated. More research had been conducted recently on vaccination in immunocompromised hosts, which could show increased efficacy and lessen vaccine delivery anxiety [25]. A study on 433 volunteers in Spain investigated the immune responses triggered by four types of COVID-19 vaccines approved by the European Medicines Agency [7]. Although the research examined recovered patients’ cellular and humoral immune responses, it did not compare responses by vaccine type. The immune responses of naive patients tended to be higher in those vaccinated with mRNA vaccines than in those vaccinated with viral vector vaccines. Several studies demonstrated that infection-acquired immunity boosted with vaccination-induced immunity remained high and could lower the likelihood of reinfection [26]. In unvaccinated individuals (defined as those without vaccination more than 14 days before symptom onset), postinfection substantial amounts of anti-RBD IgG and neutralizing antibodies were also produced. Furthermore, our study found that patients boosted with either two mRNA vaccine doses or with one AstraZeneca and one mRNA dose had superior antibody responses at 6 months postinfection than at 3 months postinfection. This finding is consistent with other studies demonstrating that heterologous regimens of COVID-19 vaccines had satisfactory effectiveness [27,28,29,30,31,32].

The waning of immunity after a SARS-CoV-2 vaccination or infection has been confirmed through various lines of evidence. Levin et al. observed a significant decrease in anti-spike IgG and neutralizing antibodies levels over the 6 months after the healthcare workers received the second dose of vaccine [32]. The decline went below the level of protection when individuals who experienced SARS-CoV-2 did not receive any COVID-19 vaccine after symptom onset. The Thai Ministry of Public Health implemented the recommendation of a COVID-19 booster vaccine after natural infection as a national health policy since late 2021 [33]. Thai citizens who have not completed the primary vaccination series are advised to receive at least the remaining dose, while individuals who have completed the primary series are recommended to have booster doses.

Several studies have confirmed that protection against severe COVID-19 illness depends on having adequate COVID-19 vaccination, provided through a primary vaccination series followed by booster doses. Thompson et al. demonstrated that three COVID-19 vaccines are highly effective in preventing severe SARS-CoV-2 infection, hospitalization, intensive care unit admission, and emergency department visits: BNT162b2 (Pfizer–BioNTech), mRNA-1273 (Moderna), and the adenovirus vector vaccine, Ad26.COV2.S (Johnson & Johnson) [27]. These vaccines provided 73% to 92% protection against emergency-department or urgent-care visits and 68% to 91% protection against hospitalization [27,34]. The vaccine effectiveness of ChAdOx1 nCoV-19 and inactivated vaccine against the symptomatic COVID-19 were 30–67% and 15.5–65.9%, respectively [35]. Some studies also reported that antibody levels were maintained longer in volunteers with a vaccine booster after the waning stage of the postinfection immune response than in individuals without a booster. Their results considered that the vaccination after recovery from COVID-19 could reduce the risk of reinfection [11,13]. Nevertheless, it is prudent to use caution when applying these findings to the recent pandemic with new variants of concerns.

COVID-19 vaccine effectiveness can be undermined by the emergence of new SARS-CoV-2 variants, such as the Omicron strain, regarding their antibody evasion properties [36]. The result of hybrid immune damping was also illustrated in a prior study [37]. Comprehensive vaccination strategies currently focus on preventing progressive diseases rather than symptomatic diseases, despite the lower infection protection ability. From the systematic review in Germany, the effectiveness in preventing any confirmed SARS-CoV-2 infection is about 6–76% and not long-lasting. With respect to severe COVID-19, the vaccine effectiveness remains high and more long-lasting, especially after receiving the booster vaccination [38]. We demonstrated that the antibody levels of unvaccinated and partially vaccinated patients who did not receive a booster vaccine decayed per the natural course of the postinfection immune response which is consistent with earlier data. Altarawneh et al. concluded that vaccination after infection could enhance protection among individuals with symptomatic Omicron infection [39]. Even if patients have not received any COVID-19 vaccine before symptom onset, having a booster regimen that uses mRNA vaccines is highly advisable for the general population. However, a booster regimen is crucial for individuals with risk factors for severe COVID-19.

The new bivalent mRNA vaccine was introduced since September 2022 to enhance the infection protection ability. A booster dose with this formulation following monovalent vaccines can provide the additional effectiveness against symptomatic SARS-CoV-2 infection [40]. However, the bivalent COVID-19 vaccine is not available in Thailand, as with other resource-limited countries. So, the administration of a monovalent mRNA vaccine for the booster dose is currently the finest possible solution for reducing the chance of reinfection and disease progression. Consequently, first generation COVID-19 vaccine boosters still have become a crucial requirement in some resource-limited countries’ recommendations for preventing disease progression, irrespective of people’s history of SARS-CoV-2 infection.

However, other clinical factors can be associated with immune responses, such as age, comorbidities, immunocompromised status, and immunosuppressive agent use. Our examination of the differences in 3- and 6-month follow-up antibody levels showed that age was inversely correlated with anti-RBD IgG levels. This finding is consistent with other studies. The multivariate analysis of a Chinese study of 192 COVID-19 patients at different hospitalization time points found correlations between IgG levels, age, and disease severity [41]. Regarding neutralizing antibodies, two studies found no association with age, which corresponded with our results [42,43]. Elderly immunogenicity has also been a source of concern since a diminished humoral response is possible. This may have increased the risk of recurrent infection that was taken into account in an earlier observational trial [11]. It is important to consider the accuracy and variance of anti-RBD IgG and neutralizing antibody levels in older individuals. Therefore, patient age is a valuable clinical predictor for COVID-19 severity and the sustainability of the humoral immune response. With declining immunity, no significant correlation between patients’ comorbidities and the variation in antibody levels could be found. The primary explanation may be because our study did not assess the severity of comorbidities, which may have had an impact on how trending the immune response was. Further studies on this topic could be required.

In COVID-19 obese patients, SARS-CoV-2 IgG antibodies have been demonstrated to be negatively correlated with BMI by Frasca et al. [44]. When compared to patients who were not reinfected, another prior retrospective study had revealed that obesity was strongly related with reinfection, with an odds ratio of 2.3 [45]. According to our findings, which included data on 40% of the reinfected participants had BMI ≥ 30 kg/m^2^, obesity appears to have an effect on the humoral immunity. Ct values, which may be acquired from several qualitative real-time polymerase chain reaction tests and are used to diagnose the majority of patients with SARS-CoV-2 infection, are connected with the amount of viral nucleic acid in a sample. Some earlier investigations demonstrated a correlation between the Ct values and clinical outcomes, such as the risk of hospitalization among COVID-19 patients and the risk of illness severity and death [46]. Although the link between Ct value and viral load is inverse, it is not linear, and a number of factors, including sample collection and PCR assay, affect this association. As a result, our observation of lower Ct values in immunocompromised patients might indicate that, although the viral load was higher, it might also be confounded by other factors, resulting in no significant association with the related clinical outcomes.

We could not discern significant differences in the immunological levels of the immunocompetent hosts, immunocompromised hosts, and reinfected patients because of their inadequate sample sizes. Furthermore, significant effects on immune responses were not observed because these patients had an unknown or mild immunocompromised status and mild degrees of comorbidity. The study cohort’s trend of decreasing antibody responses was similar to the declining response found in patients without any risk factors. Consequently, the appropriate time for a booster vaccination is 3 to 6 months postinfection. If patients do not receive a booster within 3 to 6 months, their anti-RBD IgG levels and neutralizing antibodies may substantially decay. Thus, to prolong high antibody levels, it is beneficial for patients who were unvaccinated before the onset of COVID-19 symptoms to receive a COVID-19 vaccine within 3 to 6 months after infection.

Moreover, as levels of anti-RBD IgG decrease, there is a corresponding reduction in levels of neutralizing antibodies. Several studies demonstrated a strong correlation between the levels of anti-RBD IgG and neutralizing antibodies against the Wuhan strain with previous infection or vaccination [47,48,49]. Some evidence indicates a poor correlation between anti-RBD IgG and neutralizing antibodies and the Alpha (B.1.1.7) and Beta (B.1.351) variants of SARS-CoV-2 [50,51]. The S protein mutation is among the variants of concerns that may be described. Although the level of a neutralizing antibody can provide us with more information about the protective function than the level of an anti-RBD IgG, its laboratory method was more difficult. The resulting implementation was also less common. The usage of anti-RBD IgG level may, therefore, be more applicable in real-world settings if there is any association. We assumed that the strong correlation between anti-RBD IgG and neutralizing antibodies against the Delta strain may have corresponded with the strain dominance during the pandemic. However, this finding was inconsistent with a prior Thai study that reported a highly positive correlation among neutralizing titers against all strains except Omicron [52]. This might be explained by the different methods of detecting neutralizing antibodies.

Some of our study findings need to be highlighted. First, our investigation is one of longitudinal prospective cohort studies to observe antibody responses during the 6 months after SARS-CoV-2 infection. It also analyzed Thailand’s most up-to-date data on postinfection antibody responses. Second, we evaluated three immune response categories (immunocompetent, immunocompromised, and reinfection) and analyzed their anti-RBD IgG and neutralizing antibody levels against the Wuhan and Delta strains to ascertain correlations. Third, clinical factors affecting the dynamics of the antibody responses were examined according to real-life clinical practice. We also showed that dexamethasone use and the existence of symptoms of COVID-19 did not affect the level of immune responses. Fourth, this study included subgroup analyses of various regimens of COVID-19 vaccines, including patients receiving inactivated vaccines before symptom onset. Information on inactivated vaccines is limited and is probably available only in countries such as Thailand. Fifth, this study demonstrates that routinely performing neutralizing antibody workups might not be a pragmatic approach for the general population. Our investigation found that people who acquired infection and received any COVID-19 booster regimen had neutralizing antibody levels exceeding 90%. Nevertheless, these high levels did not protect them adequately from reinfection. Last, as we did not interfere with the participants’ choices of COVID-19 vaccine, the data reflect the general population’s preferences and the disease’s natural course.

However, the results of this study must be carefully interpreted because of several limitations. First, some clinical data were incomplete because the related information was kept at other hospitals where the patients were treated. In addition, some subjects did not visit the project site on the scheduled appointment dates, particularly patients who initially attended the 1-month follow-up. Their absence made it impossible to assess immune response variations over time and to determine the antibody levels needed for protection from further symptomatic infection, especially by emerging COVID-19 variants. Moreover, we evaluated only humoral immune responses to COVID-19 infection, not any cell-mediated immune responses to infection. However, cellular immunity is also a key factor for protecting patients from developing further symptoms or a serious illness. The avidity-IgG was an alternative approach of evaluating the protective immunity that was not examined in our investigation. The likelihood of SARS-CoV-2 reinfection may be correlated with the IgG-avidity, which is defined as a measurement of the total strength of the binding between antibody and antigen. The lack of avidity maturation may have clinical significance in identifying individuals who may have a higher risk of reinfection and indicating long-term immunity [53]. This might be taken into account in future research. Lastly, although this prospective cohort study identified several significant differences between patients who had or did not have a booster dose of the COVID-19 vaccine, a randomized controlled trial would enable additional, in-depth comparisons.

## 5. Conclusions

Antibody responses (including anti-RBD IgG and neutralizing antibodies against the Wuhan and Delta strains) were maintained or increased by booster vaccinations with mRNA vaccines or heterologous vaccine regimens of mRNA and viral vector vaccines 3 to 6 months after symptom onset. In subjects with full primary vaccinations, no correlation was found between the type of vaccine prior to symptom onset or positive diagnosis. Antibody responses were higher for patients who received booster doses postinfection than those who did not. We also found strong correlations between anti-RBD IgG and neutralizing antibodies against the Delta variant. The findings are relevant to resource-limited countries for promoting the uptake of COVID-19 booster doses, particularly at 3 to 6 months after infection, to prevent progressive symptoms if reinfection occurs. The study outcomes should also aid the development of vaccination policy recommendations for patients with significant risk factors for COVID-19 in some countries, where the accessibility of bivalent vaccine is limited. According to the circulating of new variants of concerns, the implementation of this study should be carefully evaluated.

## Figures and Tables

**Table 1:** Baseline demographic and clinical characteristics of participants; *n* = 600.

Parameters	Immunocompetent (*n* = 566)	Immunocompromised (*n* = 14)	Reinfected (*n* = 20)	*p* Value
Age—years (mean ± SD)	45.8 ± 14.6	44.3 ± 10.3	37.1 ± 10.7	0.029
Sex (female)—*n* (%)	348 (61.5%)	9 (64.3%)	13 (65.0%)	0.961
BW at onset—kg (mean ± SD)	66.7 ± 15.5	60.5 ± 13.4	74.4 ± 17.7	0.029
BMI at onset—kg/m^2^ (mean ± SD)	25.4 ± 5.0	22.3 ± 4.8	27.1 ± 5.1	0.022
BW at M3—kg (mean ± SD)	67.5 ± 16.1	62.5 ± 14.4	75.3 ± 17.8	0.050
BMI at M3—kg/m^2^ (mean ± SD)	25.7 ± 5.2	23.0 ± 5.1	27.5 ± 5.0	0.048
Risk factors at onset—*n* (%)				
Age ≥ 60 years	103 (18.2%)	1 (7.1%)	1 (5.0%)	0.255
BMI ≥ 30 kg/m^2^	87 (15.4%)	1 (7.1%)	8 (40.0%)	0.015
Comorbidities	254 (44.9%)	14 (100%)	9 (45.0%)	<0.001
DM	65 (11.5%)	1 (7.1%)	3 (15.0%)	0.827
HT	145 (25.6%)	4 (28.6%)	4 (20.0%)	0.860
DLP	93 (16.4%)	6 (42.9%)	2 (10.0%)	0.038
CKD	12 (2.1%)	2 (14.3%)	0 (0.0%)	0.063
Chronic lung disease/asthma	11 (1.9%)	0 (0.0%)	0 (0.0%)	1.000
Cirrhosis	1 (0.2%)	0 (0.0%)	0 (0.0%)	1.000
ASCVD	14 (2.5%)	0 (0.0%)	0 (0.0%)	1.000
Number of comorbidities—median (IQR)	0 (0–1)	2 (1–3)	1 (0–1)	<0.001
Symptomatic infection—*n* (%)	507 (89.6%)	14 (100.0%)	19 (95.0%)	0.518
Vaccination status before infection—*n* (%)				<0.001
Unvaccinated	206 (36.4%)	8 (57.1%)	6 (30.0%)	
Partially vaccinated	211 (37.3%)	6 (42.9%)	2 (10.0%)	
Fully vaccinated	149 (26.3%)	0 (0.0%)	12 (60.0%)	
Interval between last vaccination and infection—days, median (IQR)	36.5 (16.8, 58.3)	35.5 (3.3, 61.8)	32.0 (17.0, 73.0)	0.968
Interval between onset and test—days, median (IQR)	2 (1, 4)	2 (1, 3)	2 (1, 3)	0.438
Ct value (N gene)—mean ± SD	21.5 ± 5.8	18.8 ± 3.4	21.9 ± 5.5	0.205
Favipiravir use—*n* (%)	543 (95.9%)	14 (100.0%)	20 (100.0%)	1.000
Dexamethasone use—*n* (%)	44 (8.0%)	3 (21.4%)	1 (5.3%)	0.156
Fully vaccinated at M3—*n* (%)	473 (83.6%)	12 (85.7%)	17 (85.0%)	1.000
Received any booster dose within 3 months postinfection—*n* (%)	307 (56.7%)	11 (78.6%)	6 (30.0%)	0.016
Received any booster dose between 3 and 6 months postinfection—*n* (%)	291 (65.2%)	7 (50.0%)	12 (66.7%)	0.492

ASCVD, atherosclerotic cardiovascular disease; BMI, body mass index; BW, body weight; CKD, chronic kidney disease; Ct, cycle threshold; DLP, dyslipidemia; DM, diabetes mellitus; HT, hypertension; IQR, interquartile range; N, nucleocapsid.

**Table 2:** Baseline demographic data of immunocompetent patients classified by vaccination status at 3 months postinfection; *n* = 566.

Parameters	Fully Vaccinated (*n* = 473)	Partially Vaccinated and Unvaccinated (*n* = 93)	*p* Value
Age—years (mean ± SD)	46.0 ± 14.6	44.7 ± 15.0	0.435
Gender (female)—*n* (%)	296 (62.6%)	52 (55.9%)	0.227
BW at onset—kg (mean ± SD)	66.8 ± 15.7	65.8 ± 14.7	0.562
BMI at onset—kg/m^2^ (mean ± SD)	25.5 ± 5.0	24.7 ± 5.0	0.162
BW at M3—kg (mean ± SD)	67.6 ± 16.2	67.2 ± 15.2	0.819
BMI at M3—kg/m^2^ (mean ± SD)	25.8 ± 5.2	25.2 ± 5.1	0.323
Risk factors at onset—*n* (%)			
Age ≥ 60 yr	88 (18.6%)	15 (16.1%)	0.572
BMI ≥ 30 kg/m^2^	40 (8.5%)	5 (5.4%)	0.315
Comorbidities	80 (16.9%)	11 (11.8%)	0.222
DM	212 (44.8%)	42 (45.2%)	0.952
HT	54 (11.4%)	11 (11.8%)	0.909
DLP	123 (26.0%)	22 (23.7%)	0.635
CKD	83 (17.5%)	10 (10.8%)	0.106
Chronic lung disease/asthma	10 (2.1%)	2 (2.2%)	1.000
Cirrhosis	8 (1.7%)	3 (3.2%)	0.401
ASCVD	1 (0.2%)	0 (0.0%)	1.000
Symptomatic infection—*n* (%)	422 (89.2%)	85 (91.4%)	0.529
Ct ratio (N gene)—mean ± SD	21.3 ± 5.8	22.5 ± 5.7	0.071
Favipiravir use—*n* (%)	455 (96.2%)	88 (94.6%)	0.563
Dexamethasone use—*n* (%)	37 (7.9%)	7 (8.2%)	0.926

ASCVD, atherosclerotic cardiovascular disease; BMI, body mass index; BW, body weight; CKD, chronic kidney disease; Ct, cycle threshold; DLP, dyslipidemia; DM, diabetes mellitus; HT, hypertension; N, nucleocapsid.

**Table 3:** Antibody levels of anti-RBD IgG and NAb against Wuhan and Delta strains at 3 months after SARS-CoV-2 infection in fully vaccinated participants; *n* = 158. All antibody levels are presented as median (interquartile range).

Vaccine Combination before Infection	Anti-RBD IgG (AU/mL)			Nab—Wuhan (%Inhibition)			NAb—Delta (%Inhibition)		
	No Booster Dose within 3 Months Postinfection	Booster Dose * within 3 Months Postinfection	*p* Value	No Booster Dose within 3 Months Postinfection	Booster Dose * within 3 Months Postinfection	*p* Value	No Booster Dose within 3 Months Postinfection	Booster Dose * within 3 Months Postinfection	*p* Value
SV or SP for 2 doses (*n* = 74)	12.038 (4521, 37.339)	14.542 (7579, 23,816)	0.462	96.9 (96.0, 97.6)	97.6 (97.1, 98.1)	0.004	96.6 (76.3, 98.2)	97.0 (92.5, 98.6)	0.344
AZ for 2 doses (*n* = 27)	9042 (5793, 15,558)	36,871 (19,183, 36,871)	0.051	97.4 (96.4, 97.6)	97.6 (97.5, 97.6)	0.570	94.6 (88.4, 98.3)	96.2 (93.7, 96.2)	0.462
SV or SP + AZ (*n* = 39)	7860 (4370, 15.072)	17,053 (11,350, 17.053)	0.219	96.2 (95.7, 97.2)	97.4 (97.1, 97.4)	0.113	91.8 (84.7, 97.7)	98.1 (97.3, 98.1)	0.097
SV or SP + PZ (*n* = 13)	7000 (4987, 12.343)	N/A	N/A	96.4 (96.0, 97.6)	N/A	N/A	84.2 (75.6, 97.5)	N/A	N/A
AZ + PZ (*n* = 4)	29.639 (9134, 29.639)	67,495 (67,495, 67.495)	0.500	97.5 (97.5, 97.5)	96.3 (96.3, 96.3)	0.500	98.1 (93.9, 98.1)	98.8 (98.8, 98.8)	0.500
PZ for 2 doses (*n* = 1)	3140 (3140, 3140)	N/A	N/A	91.4 (91.4, 91.4)	N/A	N/A	58.7 (58.7, 58.7)	N/A	N/A

AU, arbitrary unit; AZ, AstraZeneca; IQR, interquartile range; NAb, neutralizing antibody; N/A, not applicable (referred to no patient who received a booster dose within 3 months postinfection); PZ, Pfizer; RBD, receptor binding domain; SARS-CoV-2, severe acute respiratory syndrome coronavirus 2; SP, Sinopharm; SV, Sinovac (CoronaVac). * A booster dose included mRNA vaccines (BNT162b2, Pfizer–BioNTech) and adenovirus vector vaccine (AstraZeneca).

**Table 4:** Factors associated with immune levels. All association values were evaluated with multivariable logistic regression and are presented as median crude odds ratio (cOR) (error range) and median adjusted odds ratio (aOR) (error range); *n* = 448.

Parameters	Anti-RBD IgG not Decreased				NAb (Wuhan) not Decreased				NAb (Delta) not Decreased			
	cOR	*p* Value	aOR	*p* Value	cOR	*p* Value	aOR	*p* Value	cOR	*p* Value	aOR	*p* Value
Age	0.99	0.062	0.97	0.005	1	0.843	1.01	0.231	1	0.604	0.99	0.441
	(0.97, 1.00)		(0.95, 0.99)		(0.99, 1.02)		(0.99, 1.03)		(0.98, 1.01)		(0.98, 1.01)	
Sex (female)	1.19	0.378			0.96	0.84			1.3	0.18		
	(0.81, 1.74)				(0.65, 1.43)				(0.89, 1.91)			
BMI at 3 months	1.02	0.359			1	0.998			1.01	0.62		
	(0.98, 1.06)				(0.96, 1.04)				(0.97, 1.05)			
DM	1.13	0.7	1.54	0.386	0.6	0.152	0.6	0.223	1.01	0.986	0.94	0.894
	(0.61, 2.11)		(0.58, 4.08)		(0.30, 1.21)		(0.27, 1.36)		(0.54, 1.88)		(0.39, 2.29)	
HT	0.95	0.827			0.87	0.535			1.03	0.912		
	(0.62, 1.46)				(0.56, 1.36)				(0.67, 1.58)			
DLP	1.23	0.414	1.83	0.141	0.74	0.266	0.66	0.238	1.37	0.235	1.57	0.254
	(0.75, 2.05)		(0.82, 4.07)		(0.43, 1.26)		(0.44, 1.31)		(0.82, 2.29)		(0.72, 3.40)	
CKD	0.46	0.274	0.27	0.17	0.84	0.805	1.58	0.575	0.95	0.944	0.94	0.945
	(0.11, 1.86)		(0.04, 1.76)		(0.21, 3.40)		(0.32, 7.78)		(0.25, 3.60)		(0.16, 5.53)	
Chronic lung disease/asthma	1.88	0.47			0.84	0.841			1.54	0.622		
	(0.34, 10.35)				(0.15, 4.63)				(0.28, 8.47)			
ASCVD	1.89	0.303			1.21	0.75			1.55	0.483		
	(0.56, 6.38)				(0.38, 3.87)				(0.46, 5.21)			
Immunocompromised	0.51	0.23	0.61	0.523	0.93	0.902	1.32	0.652	1.02	0.973	1.79	0.417
	(0.17, 1.54)		(0.13, 2.77)		(0.31, 2.83)		(0.39, 4.46)		(0.35, 2.99)		(0.44, 7.34)	
Reinfection	1.9	0.248	2.39	0.299	1.49	0.447	1.68	0.361	1.55	0.431	1.76	0.414
	(0.64, 5.65)		(0.46, 12.44)		(0.53, 4.19)		(0.55, 5.12)		(0.52, 4.61)		(0.46, 6.77)	
Symptomatic infection	0.39	0.005	0.71	0.445	0.85	0.587	1.08	0.827	0.6	0.114	1.15	0.734
	(0.20, 0.75)		(0.29, 1.72)		(0.46, 1.55)		(0.54, 2.17)		(0.32, 1.13)		(0.51, 2.59)	
Ct ratio (N gene)	1.03	0.108	1.04	0.122	1.01	0.772	1.01	0.729	1.01	0.7	1.01	0.727
	(0.99, 1.06)		(0.99, 1.10)		(0.97, 1.04)		(0.97, 1.05)		(0.97, 1.04)		(0.96, 1.05)	
Favipiravir use	0.19	0.031	0.27	0.263	0.5	0.218	0.66	0.495	0.1	0.031	0.11	0.054
	(0.04, 0.86)		(0.03, 2.68)		(0.17, 1.51)		(0.19, 2.21)		(0.01, 0.81)		(0.01, 1.04)	
Dexamethasone use	0.67	0.226	1.27	0.602	0.57	0.122	0.66	0.279	0.54	0.06	0.7	0.374
	(0.35, 1.28)		(0.52, 3.12)		(0.28, 1.16)		(0.30, 1.41)		(0.28, 1.03)		(0.31, 1.55)	
Booster vaccine within 3 months but no booster vaccine between 3 and 6 months	0.5	0.247	0.49	0.239	0.31	0.008	0.29	0.005	0.45	0.058	0.42	0.046
	(0.16, 1.61)		(0.15, 1.62)		(0.13, 0.74)		(0.12, 0.68)		(0.20, 1.03)		(0.18, 0.99)	
No booster vaccine within 3 months but booster vaccine between 3 and 6 months	27.36	<0.001	29.89	<0.001	2.64	0.001	2.53	0.003	11.47	<0.001	10.83	<0.001
	(12.07, 62.02)		(12.55, 71.15)		(1.45, 4.79)		(1.37, 4.68)		(5.99, 21.95)		(5.56, 21.09)	
Booster vaccine at 0–3 months and 3–6 months	25.3	<0.001	34.05	<0.001	2.29	0.009	2.26	0.012	10.95	<0.001	11.55	<0.001
	(10.91, 58.69)		(13.88, 83.55)		(1.23, 4.28)		(1.20, 4.27)		(5.54, 21.64)		(5.74, 23.22)	

aOR, adjusted odds ratio; ASCVD, atherosclerotic cardiovascular disease; BMI, body mass index; BW, body weight; CKD, chronic kidney disease; cOR, crude odds ratio; Ct, cycle threshold; DLP, dyslipidemia; DM, diabetes mellitus; HT, hypertension; N, nucleocapsid; NAb, neutralizing antibody; OR, odds ratio; RBD, receptor binding domain.

**Table 5:** Differences in antibody responses at 3 and 6 months for different vaccine regimens. All antibody levels are presented as median (interquartile range); *n* = 18.

Vaccine Regimen	Anti-RBD IgG Difference (AU/mL)	NAb (Wuhan) Difference (%Inhibition)	NAb (Delta) Difference (%Inhibition)
2 inactivated vaccines + 1 AZ + 1 AZ (*n* = 3)	−1576 (−9668, −1576)	−0.91 (−2.95, −0.91)	−1.13 (−27.23, −1.13)
2 inactivated vaccines + 1 AZ + 1 mRNA (*n* = 7)	15.761 (11.323, 30.458)	−0.43 (−1.98, 0.63)	1.86 (1.52, 15.49)
2 inactivated vaccines + 1 mRNA + 1 mRNA (*n* = 8)	10.947 (−6674, 25.067)	−0.90 (−1.44, −0.28)	1.91 (−0.19, 6.92)
*p* value	0.117	0.788	0.788

AU, arbitrary unit; AZ, AstraZeneca; IgG, immunoglobulin G; mRNA, messenger RNA; NAb, neutralizing antibody.

**Table 6:** Antibody responses levels at 3 and 6 months of COVID-19 reinfected participants who either received or did not receive a booster dose of COVID-19 vaccine. All antibody levels are presented as median (interquartile range); *n* = 16.

Antibody Response	Anti-RBD IgG (AU/mL)			NAb—Wuhan (%Inhibition)			NAb—Delta (%Inhibition)		
	Prior Reinfection	Post Reinfection	*p* Value	Prior Reinfection	Post Reinfection	*p* Value	Prior Reinfection	Post Reinfection	*p* Value
No vaccination within 3 months postinfection (*n* = 6)	18.782 (1586, 42.055)	11.452 (5660, 16.749)	0.688	96.4 (79.2, 98.1)	96.4 (95.0, 97.1)	1.000	97.8 (18.9, 98.8)	98.2 (95.7, 98.6)	1.000
Vaccination within 3 months postinfection (*n* = 10) *	5177 (1460, 8062)	12.039 (5712, 21.486)	0.021	96.3 (92.6, 97.6)	96.9 (94.7, 97.3)	1.000	83.9 (23.4, 87.3)	98.0 (80.4, 98.5)	0.109

AU; arbitrary unit; COVID-19, coronavirus disease 2019; IgG, immunoglobulin G; NAb, neutralizing antibody. * A booster dose was administered 62 days (interquartile range, 43–75 days) before the incidence of reinfection.

## Data Availability

The data presented in this study are available on request from the corresponding author. The data are not publicly available due to privacy and ethical reasons.

## Supplementary Materials

The following supporting information can be downloaded at: https://www.mdpi.com/article/10.3390/tropicalmed8040185/s1, Table S1: Booster vaccine versus reinfection status; Table S2: Anti-RBD IgG and NAb against Wuhan and Delta strains levels in participants with 3 doses of COVID-19 vaccine at 3-month follow-up; *n* = 52. All antibody levels are presented as median (interquartile range); Table S3: Anti-RBD IgG and NAb against Wuhan and Delta strains levels for various regimens of COVID-19 vaccine in all participants; *n* = 600. All antibody levels are presented as median (interquartile range); Table S4: Anti-RBD IgG and NAb against Wuhan and Delta strains levels for various regimens of COVID-19 vaccine in immunocompetent participants; *n* = 566. All antibody levels are presented as median (interquartile range); Table S5: Anti-RBD IgG and NAb against Wuhan and Delta strains levels for various regimens of COVID-19 vaccine in immunocompromised participants; *n* = 14. All antibody levels are presented as median (interquartile range); Table S6: Anti-RBD IgG and NAb against Wuhan and Delta strains levels for various regimens of COVID-19 vaccine in participants who had reinfection; *n* = 20; Table S7: The vaccine regimen of patients who had the reinfection (*n* = 20).
