# Supplementary material for: Dynamics of Antibody Responses after Asymptomatic and Mild to Moderate SARS-CoV-2 Infections: Real-World Data in a Resource-Limited Country
Source: Trop Med Infect Dis. 2023 Mar 23;8(4):185. doi: 10.3390/tropicalmed8040185 (PMC10143231; doi:10.3390/tropicalmed8040185)
Supplement: Supplementary file 1 [file tropicalmed-08-00185-s001.zip › tropicalmed-2260206-supplementary.pdf]

Supplementary Table S1. Booster vaccine versus reinfection status

Booster vaccine	Reinfection (n=20)	OR (95% CI)
No booster dose (n = 251)	14	Reference
Boost within 3 months (n = 324)	6	0.319 (0.121–0.843)

CI, confidence interval; OR, odds ratio

Supplementary Table S2. Anti-RBD IgG and NAb against Wuhan and Delta strains levels in participants with 3 doses of COVID-19 vaccine at 3-month follow-up; n=52. All antibody levels are presented as median (interquartile range).

Regimen	Primary series	Booster dose	Anti-RBD IgG (AU/ml)	NAb - Wuhan (% inhibition)	NAb - Delta (% inhibition)
1 (n = 28)	2 Inactivated vaccines	AZ	10113 (6697, 17 401)	97.5 (97.1, 98.1)	96.5 (90.8, 97.8)
2 (n = 20)	2 Inactivated vaccines	mRNA	23099 (11 160, 36 247)	97.6 (97.0, 98.1)	98.1 (93.9, 98.7)
3 (n = 4)	At least 1 AZ	mRNA	20969 (13 308, 46 608)	97.5 (97.2, 97.7)	98.0 (94.6, 98.8)
***P* value**			0.002	0.941	0.125
P value 1 vs 2			0.002		
P value 1 vs 3			0.125		
P value 2 vs 3			1.000		

AU, arbitary unit; AZ, AstraZeneca; mRNA, messenger RNA; NAb, neutralizing antibody; RBD, receptor binding domain

Supplementary Table S3. Anti-RBD IgG and NAb against Wuhan and Delta strains levels for various regimens of COVID-19 vaccine in all participants; n = 600. All antibody levels are presented as median (interquartile range).

Group *		Anti-RBD IgG (AU/ml)			NAb – Wuhan (% inhibition)			NAb – Delta (% inhibition)		
		**1 mth**	**3 mth**	**6 mth**	**1 mth**	**3 mth**	**6 mth**	**1 mth**	**3 mth**	**6 mth**
000	n (samples)	3	63	30	3	63	30	3	63	30
	Level	445	1047	556	39.8	79.9	40.1	4.5	14.3	1.8
		(213, 445)	(547, 2932)	(279, 2472)	(26.5, 39.8)	(46.4, 95.3)	(24.2, 90.5)	(3.2, 4.5)	(3.5, 65.2)	(0, 47.9)
100	n (samples)	11	50	18	11	50	18	11	50	18
	Level	28542	9315	3569	97.9	96.9	95.6	98.5	91.1	73.3
		(17 158, 37 863)	(2841, 17 441)	(1306, 6497)	(97.7, 98.1)	(95.9, 97.5)	(84.3, 96.8)	(98.2, 98.7)	(56.9, 97.6)	(17.0, 92.9)
010	n (samples)	2	73	51	2	73	51	2	73	51
	Level	221	11275	3877	44.6	97.4	96.0	2.1	97.2	88.0
		(90, 221)	(3757, 20 043)	(1798, 7553)	(24.2, 44.6)	(95.9, 98.0)	(87.8, 96.7)	(0, 2.1)	(85.2, 98.5)	(34.1, 96.3)
110	n (samples)	4	104	72	4	104	72	4	104	72
	Level	30917	12354	4873	98.2	97.4	95.9	98.3	95.8	76.0
		(9900, 50 987)	(6100, 21 013)	(2632, 9740)	(97.7, 98.3)	(96.2, 97.9)	(92.5, 96.9)	(98.0, 98.8)	(84.4, 98.0)	(45.2, 96.5)
001	n (samples)	3	33	34	3	33	34	3	33	34
	Level	1462	771	16683	71.9	75.2	96.4	25.0	17.2	98.3
		(833, 1462)	(337, 3871)	(6216, 33 041)	(65.3, 71.9)	(42.9, 95.4)	(95.8, 97.0)	(9.5, 25.0)	(2.7, 66.8)	(91.3, 98.7)
101	n (samples)	23	129	122	23	129	122	23	129	122
	Level	37936	8097	16046	97.8	96.9	97.0	98.6	93.0	98.2
		(16 572, 52 608)	(5121, 15 821)	(9169, 25 643)	(97.5, 98.3)	(95.7, 97.6)	(96.3, 97.3)	(98.1, 98.8)	(76.5, 97.7)	(95.8, 98.7)
011	n (samples)	2	50	48	2	50	48	2	50	48
	Level	20633	7293	11053	97.8	96.9	96.3	94.5	93.0	97.1
		(18 140, 20 633)	(2809, 23 589)	(4115, 23 923)	(97.4, 97.8)	(94.5, 97.7)	(95.4, 96.9)	(91.7, 94.5)	(46.7, 98.0)	(83.0, 98.4)
111	n (samples)	8	97	96	8	97	96	8	97	96
	Level	28002	7547	22915	98.1	97.0	96.9	97.3	92.1	98.5
		(14 404, 61 772)	(4535, 16 222)	(13 017, 33 371)	(97.5, 98.3)	(95.9, 97.6)	(96.3, 97.3)	(96.0, 98.5)	(75.3, 97.2)	(97.9, 98.8)
P value		0.003	< 0.001	< 0.001	0.005	< 0.001	< 0.001	< 0.001	< 0.001	< 0.001
Total	n (samples)	56	599	471	56	599	471	56	599	471
	Level	23649	7711	11 385	97.8	96.9	96.4	98.3	91.9	97.0
		(12 104, 49 410)	(3163, 15 589)	(3868, 22 637)	(97.4, 98.2)	(95.3, 97.6)	(95.5, 97.1)	(96.0, 98.7)	(58.7, 97.7)	(77.5, 98.6)

AU, arbitary unit; IgG, immunoglobulin G; NAb, neutralizing antibody; RBD, receptor binding domain

*Group: 000, did not receive vaccine at any time; 100, received vaccine before the illness but did not receive after the illness; 010, received vaccine only within 3 months postinfection; 110, received vaccine before the illness and boost after the illness within 3 months; 001, received vaccine between 3–6 months postinfection only; 101, received vaccine before the illness and boost after the illness only at 3–6 months postinfection; 011,

did not receive vaccine before the illness and received booster vaccine 2 doses at 0–3 months and 3–6 months postinfection; 111, received vaccine all the period (before the illness, 0–3 months postinfection, 3–6 postinfection)

Supplementary Table S4. Anti-RBD IgG and NAb against Wuhan and Delta strains levels for various regimens of COVID-19 vaccine in immunocompetent participants; n = 566. All antibody levels are presented as median (interquartile range).

Group *		Anti-RBD IgG (AU/ml)			NAb – Wuhan (% inhibition)			NAb – Delta (% inhibition)		
		1 mth	3 mth	6 mth	1 mth	3 mth	6 mth	1 mth	3 mth	6 mth
000	n (samples)	3	60	27	3	60	27	3	60	27
	Level	445	1049	522	39.8	80.3	39.4	4.5	14.6	1.7
		(213, 445)	(567, 4407)	(284, 2107)	(26.5, 39.8)	(51.9, 96.1)	(25.0, 82.0)	(3.2, 4.5)	(3.8, 65.9)	(0, 38.3)
100	n (samples)	11	49	18	11	49	18	11	49	18
	Level	28542	9496	3569	97.9	96.9	95.6	98.5	91.1	73.3
		(17 158, 37 863)	(2802, 17 683)	(1306, 6497)	(97.7, 98.1)	(95.8, 97.5)	(84.3, 96.8)	(98.2, 98.7)	(55.2, 97.6)	(17.0, 92.9)
010	n (samples)	2	67	45	2	67	45	2	67	45
	Level	221	10227	3751	44.6	97.4	95.8	2.1	97.0	86.9
		(90, 221)	(3664, 19 705)	(1730, 7091)	(24.2, 44.6)	(95.9, 97.9)	(87.8, 96.4)	(0, 2.1)	(83.0, 98.5)	(33.7, 96.2)
110	n (samples)	3	99	68	3	99	68	3	99	68
	Level	46683	12458	4873	98.3	97.5	96.0	98.7	95.7	76.0
		(15 151, 46 683)	(6043, 21 031)	(2632, 9740)	(98.0, 98.3)	(96.6, 98.0)	(93.0, 96.9)	(98.0, 98.7)	(84.9, 98.0)	(45.2, 96.1)
001	n (samples)	3	31	32	3	31	32	3	31	32
	Level	1462	828	17675	71.9	76.0	96.4	25.0	20.1	98.4
		(833, 1462)	(337, 3872)	(7304, 34 096)	(65.3, 71.9)	(43.0, 96.9)	(95.8, 97.2)	(9.5, 25.0)	(3.3, 71.6)	(93.0, 98.8)
101	n (samples)	19	118	113	19	118	113	19	118	113
	Level	46160	8315	16100	97.9	96.8	97.0	98.7	94.1	98.2
		(18 532, 54 968)	(5194, 17 474)	(9398, 25 644)	(97.7, 98.3)	(95.8, 97.6)	(96.3, 97.3)	(98.2, 98.8)	(76.6, 97.8)	(96.2, 98.7)
011	n (samples)	2	47	45	2	47	45	2	47	45
	Level	20633	7676	10413	97.8	96.9	96.3	94.5	93.2	97.0
		(18 140, 20 633)	(2831, 23 127)	(4352, 22 894)	(97.4, 97.8)	(94.9, 97.7)	(95.6, 96.9)	(91.7, 94.5)	(54.1, 98.0)	(83.2, 98.3)
111	n (samples)	8	94	93	8	94	93	8	94	93
	Level	28002	7504	22518	98.1	97.0	96.9	97.3	91.8	98.5
		(14 404, 61 772)	(4536, 15 634)	(13 228, 33 319)	(97.5, 98.3)	(95.9, 97.6)	(96.3, 97.3)	(96.0, 98.5)	(74.3, 97.2)	(97.9, 98.8)
P value		0.003	< 0.001	< 0.001	0.004	< 0.001	< 0.001	< 0.001	< 0.001	< 0.001
Total	n (samples)	51	565	441	51	565	441	51	565	441
	Level	24171	7739	11385	97.8	96.9	96.4	98.3	92.2	97.0
		(11 872, 51 165)	(3255, 15 665)	(3837, 23 064)	(97.1, 98.2)	(95.4, 97.6)	(95.5, 97.1)	(95.4, 98.7)	(59.6, 97.7)	(77.1, 98.6)

AU, arbitary unit; IgG, immunoglobulin G; NAb, neutralizing antibody; RBD, receptor binding domain

*Group: 000, did not receive vaccine at any time; 100, received vaccine before the illness but did not receive after the illness; 010, received vaccine only within 3 months postinfection; 110, received vaccine before the illness and boost after the illness within 3 months; 001, received vaccine between 3–6 months postinfection only; 101, received vaccine before the illness and boost after the illness only at 3–6 months postinfection; 011, did not receive vaccine before the illness and received booster vaccine 2 doses at 0–3 months and 3–6 months postinfection; 111, received vaccine all the period (before the illness, 0–3 months postinfection, 3–6 postinfection)

Supplementary Table S5. Anti-RBD IgG and NAb against Wuhan and Delta strains levels for various regimens of COVID-19 vaccine in immunocompromised participants; n = 14. All antibody levels are presented as median (interquartile range).

**Group***		**Anti-RBD IgG (AU/ml)**			**NAb – Wuhan (% inhibition)**			**NAb – Delta (% inhibition)**		
		**1 mth**	**3 mth**	**6 mth**	**1 mth**	**3 mth**	**6 mth**	**1 mth**	**3 mth**	**6 mth**
000	n (samples)		1	1		1	1		1	1
	Level		35	22		28	0		0	0
			(35, 35)	(22, 22)		(28, 28)	(0, 0)		(0, 0)	(0, 0)
100	n (samples)									
	Level									
010	n (samples)		4	4		4	4		4	4
	Level		12346	6706		97.3	96.9		96.3	92.2
			(5322, 63 103)	(2531, 10 651)		(91.4, 97.8)	(87.3, 97.1)		(64.6, 98.6)	(44.6, 93.8)
110	n (samples)		2	2		2	2		2	2
	Level		5067	2773		81.7	80.9		60.8	37.1
			(3486, 5067)	(1125, 2773)		(68.4, 81.7)	(73.9, 80.9)		(48.6, 60.8)	(17.5, 37.1)
001	n (samples)		1	1		1	1		1	1
	Level		474	13725		49.5	96.9		5.0	98.2
			(474, 474)	(13 725, 13 725)		(49.5, 49.5)	(96.9, 96.9)		(5.0, 5.0)	(98.2, 98.2)
101	n (samples)		1	1		1	1		1	1
	Level		2436	4576		90.3	97.0		25.5	86.3
			(2436, 2436)	(4576, 4576)		(90.3, 90.3)	(97.0, 97.0)		(25.5, 25.5)	(86.3, 86.3)
011	n (samples)		2	2		2	2		2	2
	Level		14761	13738		74.9	93.1		53.1	74.3
			(314, 14 761)	(2037, 13 738)		(51.4, 74.9)	(90.0, 93.1)		(7.55, 53.1)	(49.9, 74.3)
111	n (samples)		3	3		3	3		3	3
	Level		22706	24047		97.5	96.9		96.1	98.6
			(4418, 22 706)	(7753, 24 047)		(95.2, 97.5)	(96.6, 96.9)		(79.4, 96.1)	(94.1, 98.6)
Total	n (samples)		14	14		14	14		14	14
	Level		5533	6706		95.1	96.7		76.2	92.2
			(1945, 22 966)	(1866, 16 305)		(64.1, 97.6)	(87.0, 96.9)		(21.0, 97.7)	(44.7, 98.3)

AU, arbitary unit; IgG, immunoglobulin G; NAb, neutralizing antibody; RBD, receptor binding domain
*Group: 000, did not receive vaccine at any time; 100, received vaccine before the illness but did not receive after the illness; 010, received vaccine only within 3 months postinfection; 110, received vaccine before the illness and boost after the illness within 3 months; 001, received vaccine between 3–6 months postinfection only; 101, received vaccine before the illness and boost after the illness only at 3–6 months postinfection; 011, did not receive vaccine before the illness and received booster vaccine 2 doses at 0–3 months and 3–6 months postinfection; 111, received vaccine all the period (before the illness, 0–3 months postinfection, 3–6 postinfection)
**Difference between groups can't be evaluated due to small sample size

Supplementary Table S6. Anti-RBD IgG and NAb against Wuhan and Delta strains levels for various regimens of COVID-19 vaccine in participants who had reinfection; n = 20

**Group***		**Anti-RBD IgG (AU/ml)**			**NAb – Wuhan (% inhibition)**			**NAb – Delta (% inhibition)**		
		**1 mth**	**3 mth**	**6 mth**	**1 mth**	**3 mth**	**6 mth**	**1 mth**	**3 mth**	**6 mth**
000	n (samples)		2	2		2	2		2	2
	Level		1172 (345, 1172)	7211 (3029, 7211)		66.4 (40.9, 66.4)	95.5 (93.4, 95.5)		12.6 (0, 12.6)	94.0 (90.0, 94.0)
100	n (samples)		1			1			1	
	Level		9134 (9134, 9134)			97.5 (97.5, 97.5)			93.9 (93.9, 93.9)	
010	n (samples)		2	2		2	2		2	2
	Level		44953 (39 157, 44 953)	14792 (11 511, 14 792)		98.1 (98.1, 98.1)	96.4 (96.4, 96.4)		98.7 (98.7, 98.7)	98.5 (98.3, 98.5)
110	n (samples)	1	3	2	1	3	2	1	3	2
	Level	8150 (8150, 8150)	14808 (11 652, 14 808)	11422 (6537, 11 422)	97.6 (97.6, 97.6)	97.1 (95.8, 97.1)	96.2 (95.5, 96.2)	98 (98, 98)	96.9 (95.9, 96.9)	98.1 (97.6, 98.1)
001	n (samples)		1	1		1	1		1	1
	Level		122 (122, 122)	2256 (2256, 2256)		32.3 (32.3, 32.3)	91.4 (91.4, 91.4)		2.0 (2.0, 2.0)	23.7 (23.7, 23.7)

101	n (samples)	4	10	8	4	10	8	4	10	8
	Level	26585 (14 811, 46 446)	5922 (4233, 8963)	14508 (6958, 28 557)	97.6 (97.5, 97.7)	97.1 (94.7, 97.6)	96.9 (95.8, 97.2)	98.2 (98.1, 98.5)	85.4 (73.2, 87.3)	98.0 (92.6, 98.5)
011	n (samples)		1	1		1	1		1	1
	Level		1314 (1314, 1314)	11704 (11 704, 11 704)		94.6 (94.6, 94.6)	97.7 (97.7, 97.7)		13.8 (13.8, 13.8)	98.6 (98.6, 98.6)
111	n (samples)									
	Level									
Total	n (samples)	5	20	16	5	20	16	5	20	16
	Level	16572 (11 187, 43 163)	6810 (2342, 13 581)	11607 (6619, 17 624)	97.6 (97.5, 97.7)	97.0 (93.4, 97.6)	96.6 (95.6, 97.2)	98.1 (98.0, 98.4)	85.8 (36.6, 96.7)	98.1 (92.6, 98.5)

AU, arbitary unit; IgG, immunoglobulin G; NAb, neutralizing antibody; RBD, receptor binding domain

*Group: 000, did not receive vaccine at any time; 100, received vaccine before the illness but did not receive after the illness; 010, received vaccine only within 3 months postinfection; 110, received vaccine before the illness and boost after the illness within 3 months; 001, received vaccine between 3–6 months postinfection only; 101, received vaccine before the illness and boost after the illness only at 3–6 months postinfection; 011, did not receive vaccine before the illness and received booster vaccine 2 doses at 0–3 months and 3–6 months postinfection; 111, received vaccine all the period (before the illness, 0–3 months postinfection, 3–6 postinfection)

**Difference between groups can’t be evaluated due to small sample size

Supplementary Table S7. The vaccine regimen of patients who had the reinfection (n=20)

Group*	n	Vaccine prior to first infection onset	Vaccine within 3 months postinfection	Vaccine between 3 and 6 months postinfection
000	2	no	no	no
100	1	AZ + PZ	no	N/A
010	2	no	PZ	no
110	1	SP + SP	PZ	no
	1	SV + SV + AZ	PZ	no
	1	AZ	AZ	N/A
001	1	no	no	AZ
101	1	SV + SV	no	PZ
	1	SP + SP	no	AZ
	5	SV + SV + AZ	no	PZ
	2	AZ + AZ	no	PZ
	1	AZ + AZ	no	AZ
011	1	no	SV	AZ
111	0	N/A	N/A	N/A

AZ, AstraZeneca; N/A, not available; PZ, Pfizer; SP, Sinopharm; SV, Sinovac (CoronaVac)

*Group: 000, did not receive vaccine at any time; 100, received vaccine before the illness but did not receive after the illness; 010, received vaccine only within 3 months postinfection; 110, received vaccine before the illness and boost after the illness within 3 months; 001, received vaccine between 3–6 months postinfection only; 101, received vaccine before the illness and boost after the illness only at 3–6 months postinfection; 011, did not receive vaccine before the illness and received booster vaccine 2 doses at 0–3 months and 3–6 months postinfection; 111, received vaccine all the period (before the illness, 0–3 months postinfection, 3–6 postinfection)
